# Evaluation of optimized continuous venovenous hemodiafiltration therapy efficiency in severe burn patients with sepsis

**DOI:** 10.4103/2321-3868.137604

**Published:** 2014-07-28

**Authors:** Cheng Xu, Kunwu Fan, Lihua Xie, Wanan Chen, Liya Wang

**Affiliations:** 1 Department of Burn and Plastics, Second Hospital of Shenzhen, Guangdong, 518035 China; 2 Emory University School of Medicine, Atlanta, Georgia 30322 USA

**Keywords:** Burn, inflammatory, sepsis, continuous vein-vein hemodiafiltration

## Abstract

As an initial factor, sepsis and multiple organ dysfunction syndrome (MODS) caused by sepsis are the principal causes of death in burned patients. In this report, we measured the levels of tumor necrosis factor (TNF)-α, interleukin (IL)-6 and IL-8 in severely burned patients with sepsis after the initiation of continuous vein-vein hemodiafiltration (CVVHDF) to evaluate the clinical usefulness of CVVHDF on the removal of key mediators. The vital sign indices, such as the heart rate (HR), respiration (R) and central venous pressure (CVP), were recorded at 0 and 42 h in each group. Further, the laboratory examinations indexes, such as the white blood cell count, blood sugar, serum sodium, blood urea nitrogen and serum creatinine, were detected in venous blood samples. Twenty-two severely burned patients suffering from sepsis were randomized into the control group (A, *n* = 11) and the experimental group (B, *n* = 11). The patients in group A underwent conventional treatment, and those in group B received conventional+CVVHDF treatment. The vital signs, such as the HR, R, and CVP, and laboratory examination indices, such as the blood cell count, blood sugar, serum sodium, blood urea nitrogen, and serum creatinine, dropped significantly in group B compared with those in group A at 42 h (*P* < 0.05). The plasma levels of TNF-α, IL-6 and IL-8 were measured at 0, 12, 18, 24, 36 and 42 h after the start of CVVHDF and at the same time points after the patients were diagnosed with sepsis in group A. The plasma levels of TNF-α in group B decreased by 32% at 18 h after the start of CVVHDF and decreased by 43% at 42 h after the start of CVVHDF; however, these levels were increased compared with the normal values (*P* < 0.01). The plasma levels of IL-6 decreased at 18 h after the start of CVVHDF (0.274 ± 0.137 ng/ml). Following a brief increase at 24 h, the plasma levels of IL-6 again decreased continuously until the end of the investigation (0.192 ± 0.119 ng/ml). The plasma levels of IL-8 in group B decreased by 56% at 18 h after the start of CVVHDF, but they were increased compared with the normal values (*P* < 0.01). The plasma levels of IL-8 in group B decreased by 70% at 42 h after the start of CVVHDF, but they were increased compared with the normal values (*P* < 0.01). The MODS incident was 4 of 11 in group A compared with 1 of 11 in group B (*P* < 0.01). In conclusion, CVVHDF can effectively reduce the levels of TNF-α, IL-6 and IL-8 as well as the MODS incidence in patients with serious burns.

## Introduction

Sepsis is a major complication of severe burn and is one of the principal causes of death in burned patients. As an initial factor, sepsis and multiple organ dysfunction syndrome (MODS) caused by sepsis are the principal causes of death in burned patients. Despite the impressive advances in our understanding of the basic mechanisms of sepsis, the mortality rates in burned patients that are associated with sepsis remain high. In 1992, sepsis was considered a systemic inflammatory response syndrome (SIRS) that is caused by a serious infection[1], and this definition of sepsis is still used today. The foundation of the clinical pathophysiology of sepsis is SIRS while the etiology resulting in SIRS with the sepsis status is infection.[2] Sepsis due to bacterial infection or SIRS due to non-infectious causes, such as trauma like burns, develops in part from the release of many biologically active inflammatory mediators. Great importance has been attached to the study of treating sepsis after burns worldwide. Continuous vein-vein hemodiafiltration (CVVHDF) can effectively eliminate the endogenous and exogenous toxins and regulate homeostasis. Thus, CVVHDF can become an effective treatment option for sepsis. From June 2003 to October 2012, burned patients with sepsis were treated with optimized CVVHDF in our working unit, and good clinical effects have been achieved.

**Table:** 

Access this article online	
**Quick Response Code**:	**Website**: www.burnstrauma.com
	**DOI**: 10.4103/2321-3868.137604

## Objective and methods

### General data

Twenty-two severely burned patients with sepsis, including 19 males and 3 females, ranging in age from 19 to 46 years, were recruited in this study. These 22 patients included flame burn (*n* = 16), chemical burn (*n* = 3), and hydrothermal burn (*n* = 3) patients. Twenty-two patients were randomized into group A (*n* = 11) and group B (*n* = 11). Patients in group A received conventional treatment, and patients in group B received conventional + CVVHDF treatment. They all were from the burn centre in the Second People Hospital, Shenzhen, Guangdong, China from June 2003 to October 2012. The criteria for diagnosing sepsis and MODS were based on the clinical and laboratory findings.[3,4] The diagnosis of sepsis was based on evidence aetiology or cases that were highly suspected to have infection; additionally, patients who conformed to the any 2 of the first 4 items of the following list plus any single part of item 5 were diagnosed as having sepsis:

1. Temperature > 39.0°C or < 35.5°C, continuous 3 d;
2. Heart rate > 120 beats per minute;
3. WBC > 12.0 × 10^9^/l or < 4.0 × 10^9^/l, including neutrophils > 80%;
4. Breathing rate > 28 times/min; and
5. Clinical burn symptoms such as depression, irritability or delirious speech; abdominal distension, diarrhea or gastrointestinal bleeding; and tongue color changes, burr, dry and without saliva.

Informed consent was obtained from the patients or their family members according to the guidelines of the Ethics Committee.

### Protocol

Subjects in the experimental group were treated with CVVHDF using the Prisma system (BRAUN Diapact, Japan); a dual lumen central venous catheter (17 cm BRAUN Haemocat Signo, USA) was usually inserted in non-burned areas, most commonly into the femoral vein, or rarely into the external jugular veins. The CVVHDF was conducted near the bed; the displacement liquid was Port formula, and the blood flow rate was maintained at between 80 and 100 ml/min. The displacement fluid was infused pre-dilution at a rate varying between 1,500 and 2,000 ml/h, and each treatment lasted 12 h.

### Measurements

Venous blood samples were drawn into pyrogen-free heparinized blood specimen tubes at 0, 12, 18, 24, 36 and 42 h after CVVHDF initiation in group B and after the patients were diagnosed with sepsis in group A. They were immediately centrifuged at 3,000 rpm (the centrifugal radius was 13.5 centimetres) for 15 min, and plasma was obtained and stored at −20°C until the mediator assays were performed. Samples (0.5 ml) of ultrafiltrate were collected in pyrogen-free vials at 0, 12, 18, 24, 36 and 42 h after CVVHDF initiation and frozen at −20°C until the assay. According to the manufacturer’s instructions, the tumor necrosis factor (TNF)-α, interleukin (IL)-6 and IL-8 levels in the plasma and ultrafiltrate were detected using an enzyme linked immunosorbent assay (the kits were provided by Boster Biological Engineering Co., Ltd, Wuhan). The vital sign indices, such as heart rate (HR), respiration (R) and central venous pressure (CVP), were recorded at 0 and 42 h in each group. The laboratory examination indices, such as the white blood cell count, blood sugar, serum sodium, blood urea nitrogen and serum creatinine, were also detected in the venous blood samples.

### Statistical analysis

Measurement data are presented as the mean ± standard error , and the enumeration data are presented as the constituent ratio. The statistical analysis of the measurement data was performed using two-way repeated measures analysis of variance (ANOVA), and that of the enumeration data was performed using the chi-square test with the Statistical Package for the Social Sciences software (SPSS 19.0). Differences with *P* < 0.05 were considered significant.

## Results

### Patient demographics

The differences in gender, age, total burn surface area (TBSA) and burn depth between group A and B were not statistically significant (*P* > 0.05) [Table 1].

**Table 1:** **Parameters at the time of admission for both groups**

Group	Number	Gender (male/female)	Age (years)	BSA (%)		
				TBSA (%)	II° burn (%)	III° burn (%)
group A	11	10/1	31.4±11.1	66.71±19.73	36.93±14.82	19.79±8.23
group B	11	9/2	31.1±9.1	60.94±19.26	35.81±20.23	11.45±6.54
*P* value		1.000	0.932	0.463	0.868	0.394

BSA = burn surface area, TBSA = total burn surface area

### Changes of vital sign indices

Compared with the vital sign indices at 0 h, these indices decreased slightly by 42 h in group A. There were no significant differences (*P* > 0.05). However, compared with the HR, R and CVP indices at 0 h, these indices obviously improved by 42 h in group B (*P* < 0.05). Compared with group A at 42 h, the HR, R and CVP indices in group B decreased significantly by 42 h (*P* < 0.05) [Table 2].

**Table 2:** **Changes in the vital signs for both groups**

Group	Number	Time point	HR (beats/min)	R (times/min)	CVP (cmH_2_O)
group A	11	0 h	155.3±36.2	38.3±13.6	13.7±2.9
		42 h	144.1±38.9	32.8±12.4	14.1±5.2
group B	11	0 h	154.6±40.1	34.5±13.6	13.6±4.8
		42 h	126.8±35.7^ab^	25.3±11.2^ab^	11.3±2.5^ab^

^a^denotes the result of the comparison between 0 and 42 h in group B, *P* < 0.05, ^b^denotes the result of the comparison between groups A and B at the corresponding time point, *P* < 0.05

### Changes in laboratory examination indices

The laboratory examination indices, including the white blood cell count, blood sugar, serum sodium, blood urea nitrogen and serum creatinine, at 42 h in group B obviously decreased (*P* < 0.05) compared with these clinical indices at 0 h. However, in group A at 42 h, the white blood cell count, blood sugar, serum sodium, blood urea nitrogen and serum creatinine dropped slightly compared with these clinical indices at 0 h. This difference had no statistical significance (*P* > 0.05). The laboratory examination indices, including the blood cell count, blood sugar, serum sodium, blood urea nitrogen, and serum creatinine in group B dropped significantly compared with these indices in group A at 42 h; this difference is statistically significant (*P* < 0.05) [Table 3].

**Table 3:** **Changes in the laboratory examination indices in both groups**

Group	Number	Time point	Na (mmol/L)	BUN (mmol/L)	Scr (µmol/L)	BS (mmol/L)	WBC (× 10^9^)
group A	11	0 h	143.88±7.09	19.11±3.36	102.46±18.33	12.81±3.04	21.36±3.62
		42 h	139.46±5.84	16.23±4.15	96.98±16.91	11.66±1.92	18.64±2.29
group B	11	0 h	144.89±7.36	18.53±3.72	106.85±13.38	13.17±3.85	20.63±3.84
		42 h	132.83±6.16^ab^	12.91±4.47^ab^	92.06±15.03^ab^	8.69±2.61^ab^	10.82±1.37^ab^

^a^denotes the result of the comparison between 0 and 42 h in group B, *P* < 0.05, ^b^denotes the result of the comparison between groups A and B at the corresponding time point, *P* < 0.05

### Changes in plasma TNF-α

**Figure 1:**
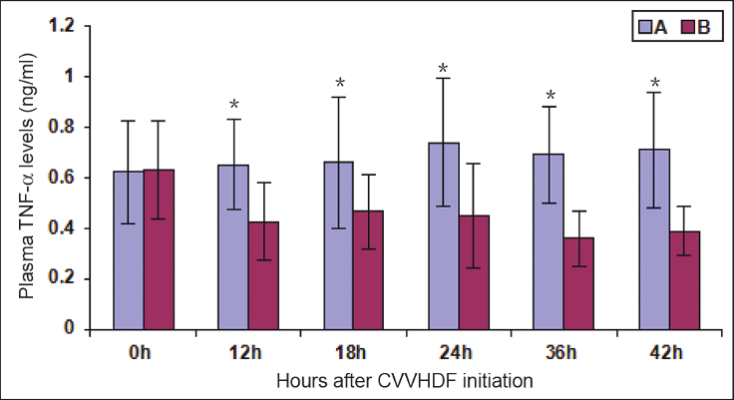
Changes in plasma tumor necrosis factor (TNF)-α after continous vein-vein hemodiafiltration (CVVHDF) initiation ^‘*’^denotes the result of the comparison between groups A and B at the corresponding time point, *P* < 0.01.

The plasma levels of TNF-α exhibited no significant differences between group A (0.625 ± 0.202 ng/ml) and group B (0.634 ± 0.193 ng/ml) before the CVVHDF initiation. The plasma levels of TNF-α in group B were decreased by 32% at 18 h after the start of CVVHDF, but they were higher than the normal values (*P* < 0.01). The TNF-α levels in group B decreased by 43% at 42 h after the start of CVVHDF, but they were increased compared with the normal values (*P* < 0.01) [Figure 1].

### Changes in plasma IL-6

**Figure 2:**
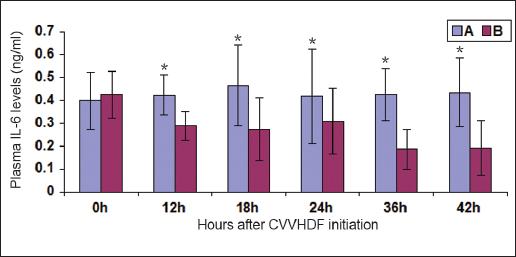
Changes in plasma interleukin (IL)-6 after continous vein-vein hemodiafiltration (CVVHDF) initiation ^‘*’^denotes the result of the comparison between groups A and B at the corresponding time point, *P* < 0.01.

There were no significant differences in the plasma levels of IL-6 between group A (0.399 ± 0.126 ng/ml) and group B (0.426 ± 0.102 ng/ml) before the initiation of CVVHDF. The plasma levels of IL-6 in group B decreased at 18 h after the start of CVVHDF (0.274 ± 0.137 ng/ml). Following a brief increase in group B at 24 h (0.309 ± 0.144 ng/ml), the plasma levels of IL-6 again decreased continuously until the end of the investigation (0.192 ± 0.119 ng/ml) [Figure 2].

### Changes in plasma IL-8

**Figure 3:**
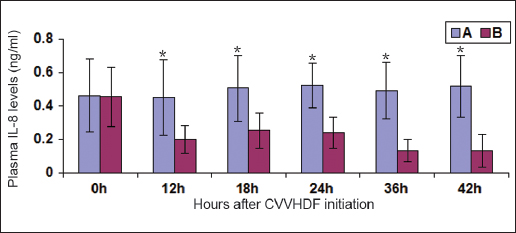
Changes in plasma interleukin (IL)-8 after continous vein-vein hemodiafiltration (CVVHDF) initiation ^‘*’^denotes the result of the comparison between groups A and B at the corresponding time point, *P* < 0.01.

There were no significant differences in the plasma IL-8 levels between group A (0.462 ± 0.218 ng/ml) and group B (0.455 ± 0.176 ng/ml) before the initiation of CVVHDF. The plasma levels of IL-8 in group B were decreased by 56% at 18 h after the start of CVVHDF, but they were increased compared with the normal values (*P* < 0.01). The plasma levels of IL-8 in group B were decreased by 70% at 42 h after the start of CVVHDF, but they were increased compared with the normal values (*P* < 0.01). There were significant differences in the changes in the plasma IL-8 levels between the groups during the study interval (*P* < 0.01) [Figure 3].

### Outcomes and complications

MODS occurred in 4 of the 11 patients in group A compared with 1 of the 11 patients in group B (*P* < 0.01). No differences in the median stay in the ICU or total days in the hospital were found between the groups. No differences in mortality were found between the groups, although there were 2 deaths in group A versus 1 in group B. There were no heparin-related complications or other CVVHDF-related complications in any of the subjects in this study.

## Discussion

Due to the early stress response and burn tissue necrosis in severe burn patients, the endotoxin that comes from the wounds and intestinal tracts causes the release of inflammatory mediators. Substantial inflammatory mediators would cause SIRS, which may be constantly exacerbated through the cascade effect and give rise to the “waterfall effect” of inflammation[5], which could in turn lead to multiple organ injury and, eventually, MODS.

Continuous renal replacement therapy (CRRT) technology is a new technology, and it has been widely applied to rescue patients with multiple organ failure, which has improved the survival rate of critical cases. CVVHDF is a type of CRRT that was first applied to treat patients with acute renal insufficiency that is unstable in hemodynamics. According to the research, CRRT can eliminate the inflammatory mediator in extracorporeal circulation and improve the prognosis of sepsis and MODS in animal experiments.[6] Furthermore, its effectiveness is related to the treatment strength and method.[7] CVVHDF has already been widely applied to treat acute severe patients.

In the early stage of burn sepsis, CVVHDF could improve organ function and prevent MODS. Its mechanism may be the following:

1. CVVHDF eliminates the inflammatory medium and cell factors, such as IL-6, IL-8, IL-1β and TNF-α, to interrupt the progress of SIRS towards MODS and MOF; and
2. CVVHDF improves the cardiopulmonary function. CVVHDF can eliminate some toxins that could injure the cardiopulmonary function, perfect the lung function of gas exchange, correct the volume load and alleviate the cardiac preload.

The severity of multi-organ function injury and case fatality rate are positively correlated with the plasma levels of inflammatory medium[8], and IL-6 is the main member of the cytokine network, possibly playing a significant role in various infectious diseases. Additionally, it is a marking signal that reflects the inflammatory response of the body. Particularly in such pathological states as sepsis and SIRS, it can detect increasing IL-6 levels, and its level is closely related to the degree of infection and prognosis.[9] TNF-α is an endogenous cell regulatory factor that is generated by the activated mononuclear macrophage system; it is also the main factor of immediate inflammatory reactions. Currently, TNF-α is thought to come from burn patients’ Kupffer cells, which are found in the liver. TNF-α has a dual-directional regulation function, and only a small dose of TNF-α can enhance the immunity of the burn patient. A continuous and high level of TNF-α could hint at a poor prognosis of severe infection.[10,11]

Burn patients with sepsis in our hospital were treated with CVVHDF for 12 h, and the blood flow rate was maintained at between 80 and 100 ml/min. The treatment lasted for 42 h. According to the research results, CVVHDF can effectively reduce the content of various inflammatory factors, thereby decreasing the incidence rate of MODS. Further, CVVHDF treatment can effectively decrease the laboratory examination indices, such as the white blood cell count, blood sugar, serum sodium, blood urea nitrogen and serum creatinine. This can improve the environment in the body of severe burn patients with sepsis. Therefore, CVVH treatment is more effective in the primary stage of sepsis. In future research, we will attempt to adopt larger samples for further research to obtain more scientific evidence for the treatment of burn sepsis with CVVHDF.

## Reasons for Submission to Burns & Trauma

### About the Journal

Burns & Trauma, official journal of Chinese Burn Association, is a peer-reviewed online journal published quarterly. The journal publishes most innovative and creative studies in the field of burns and trauma, and allows free access (Open Access) to its contents.

- Fast peer review process
- Broad view by OPEN ACCESS
- No page charges
- Provide Language polishing service
- Advanced online publication
- Awards for excellent articles

Full texts are available on http://www.burnstrauma.com, or iPad app of Burns & Trauma.
